# The effect of reinforcement on the mechanical properties of veneered wood fiber/polypropylene composites assembled with chlorinated polypropylene

**DOI:** 10.1038/s41598-022-17777-w

**Published:** 2022-08-17

**Authors:** Yinan Liu, Feng Chen, Xiaohui Ni, Xinghua Xia

**Affiliations:** 1 School of Art and Design, Taizhou University, Taizhou, 318000 People’s Republic of China; 2 Faculty of Creative Technology and Heritage, University Malaysia Kelantan, 16100 Kelantan, Malaysia

**Keywords:** Structural materials, Techniques and instrumentation

## Abstract

Wood fiber/polypropylene (WF/PP) composites are environmentally friendly materials with high dimensional stability and mechanical properties. However, the applications of WF/PP composites are limited by an unattractive surface texture. In this study, the WF/PP composites were decorated with poplar wood veneer using chlorinated polypropylene (CPP) as a hot melt adhesive, the bonding strength is over 1.18 MPa. Surface bonding strength tests and scanning electron microscopy (SEM) were performed to analyze the quality of the CPP bonding layer. The physical and mechanical properties of the veneered WF/PP boards and unveneered controls were examined to determine the effects of reinforcement. The result shows that after veneered the tension strength and modulus of the whole composite board were increased over 30% and 10%; the flexural strength and modulus were increased over 10% and 20%. Low-velocity impact testing was performed to determine the impact resistance properties of the composites. Higher ratios of wood fiber in the WF/PP composite led to a higher surface bonding strength, which was evident in the SEM images. Wood veneer decoration increased the mechanical strength of the whole composite board. A tighter bond along the CPP layer would provide additional reinforcement of the veneered composite’s mechanical properties.

## Introduction

Wood fiber/polypropylene (WF/PP) composite materials are one of several wood plastic composites (WPCs). The WF/PP composite has high physical and mechanical properties even the PP within matrix is recycled^1^. Wood plastic composites are made by combining dry plant material (typically wood, bamboo, hemp, and straw) with a polymer matrix with a small number of additional additives such as bonding improvers like maleic anhydride bonded polypropylene (MAPP), which bonds to both wood fibers, and PP. The addition of such additives improves the quality of the WP/PP composites. Thermoplastics are the most common type of polymer matrix. Thermoplastic WPCs are commonly formed into products by extrusion, mold pressing, and injection molding in ways that do not considerably differ from the methods by which unfilled thermoplastics are formed into products^2,3^. Many different thermoplastics can be used to make WPCs, including recycled plastics^4–6^.

A major advantage of thermoplastic WPCs is that no adhesives, resins, or other volatile chemical reagents are typically required. Thermoplastic WPCs are a comparatively non-toxic and environmentally friendly material^7^. Wood plastic composites combine many of the best features of plastics and wood. A typical WPC will have higher mechanical strength properties and better dimensional stability compared to an unfilled thermoplastic of the same type that is being used for the matrix. In addition, a typical WPC will possess greater resistance to moisture and decay than the plant material that is being added to the thermoplastic. Wood plastic composites are much easier to process and mold than wood^8–10^. In recent years, WPCs have been used in many applications such as outdoor facilities, automobile linings, and exterior wall cladding^11–15^. However, current processing techniques leave the surface texture of WPCs aesthetically unsatisfactory for any purpose in which the WPC will be subjected to close visual attention, such as visible indoor surfaces. If the surface of a WPC can be reliably and durably bonded to a wooden veneer, the field of applications will considerably increase. Many of the new WPC products, as well as high-value products, are made possible by veneering.

In the process of extrusion and hot pressing, a thin layer of unfilled plastic matrix will gather on most areas of the WPC surface^16,17^. This surface of comparatively pure plastic will not disappear after cooling and shaping. This plastic surface is smooth, without pores, and it bonds poorly with the room temperature adhesives that are used in traditional veneering processes^18^. The essential problem is that most forms of plastic are chemically inert and extremely non-polar to form strong bonds with common adhesives at room temperature. Neither covalent nor polar (hydrogen) bonds are likely to form between the plastic and the adhesive unless something is done to promote this type of bonding.

To improve the bond between the plastic and the adhesive at room temperature, plastics often have their surfaces modified with varying degrees of success. There are several common surface modification techniques for plastics to improve adhesive bonding. Strongly oxidizing acids or other oxidizing agents can be used to treat the surface of composite materials and to produce a certain etching effect on the surface and generate polar groups which increase the plastic’s compatibility with polar groups on the adhesive molecules. Surface modification with a plasma discharge can be used to create surface roughness and polar groups^19,20^. A chemical coupling agent can also be used to form a covalently bonded chemical bridge between the plastic molecules and the adhesive molecules. Lastly, there is mechanical roughening, which works by magnifying the area wetted by the adhesive and lengthening the bond line at the microscopic level. Although such methods can increase the bonding effect of adhesives on the surface of plastics and WPCs, there are many associated issues and difficulties such as poor environmental outcomes, complicated treatment processes, and high costs.

For the bonding of thermoplastics to other thermoplastics, the most straightforward method is to use room-temperature glues and surface modification and weld the pieces together with molten thermoplastic of a chemically compatible nature. This process is similar to the welding, brazing, or soldering of metals.

Molten thermoplastics, with additives to promote adhesion to natural fibers, can be used to join pieces of wood and other cellulosic materials such as cardboard to each other in industrial processes such as the manufacturing of boxes, where the rapid cooling and hardening of the thermoplastic keeps production flowing rapidly along. In these applications, the molten thermoplastic is referred to as a hot melt adhesive to distinguish it from room temperature adhesives.

Furthermore, it follows that it is possible to successfully bond wood veneer to a plastic substrate using hot melt adhesives. For example, wood veneer can be successfully bonded to unfilled PP and to WF/PP, the subject of this study, using MAPP as the hot melt adhesive. Unfortunately, although thermoplastics melt at lower temperatures than most metals, the temperature of hot melt adhesive is still extremely high in most cases that damage to the veneer surface. The hot melt working temperature of MAPP is 170 °C. This is still high enough to change the color of wood surfaces and generally degrade the performance of a wood veneer.

For the purposes of this study, chlorinated polypropylene (CPP) was selected as a hot melt adhesive for joining wood veneer to WF/PP. Chlorinated polypropylene has a considerably lower melting point than MAPP. Chlorinated polypropylene has an initial melting point of 90 °C and it becomes sufficiently fluid for hot melt processing at 110 °C. At 110 °C, the original color and characteristics of the surface veneer is preserved. Furthermore, there is less risk of dimensional changes in the underlying WF/PP material. The use of CPP for this purpose has already been reported by Liu et al*.*^21^. This study reported the mechanical properties of CPP-bonded veneer laminates on WF/PP for indoor and outdoor furniture uses. Moreover, for flooring, it is important that the veneered product has adequate bending, tensile, and impact strength characteristics. These parameters tend to change with temperature, so they were investigated across a range of temperatures.

## Material and methods

### Materials

Poplar wood veneers (thickness of 1.5 ± 0.1 mm) of identical grade were obtained from Jinan Yuanfang Wood Trading Company (Jinan, China). A portion of these veneers was randomly selected to be used as veneers for this experiment, other wood veneers were grounded into wood fibers which were filtered with 40 mesh filter gauze for incorporation into the WF/PP composites. The PP (T300; Sinopec Daqing Petrochemical Co., Daqing, China) had a melting point of 168 °C, a density of 0.91 g/L, and a melt flow rate (MFR) of 0.25 to 0.35 g/min at 180 °C. The MAPP (Shanghai Sunny New Technology Development Co., Shanghai, China) had a grafting percentage of 1% to 1.2%. The CPP (Shenzhen Jitian Chemical Products Limited Co., Shenzhen, China) was obtained as pellets with a chlorination ratio of 32%, a MFR of 1.66 g/min to 2.01 g/min at 110 °C, a melting point of 90 °C, and a density of 0.93 g/L.

### Preparation of the WF/PP composites

The WFs were dried by an impulse-cyclone drying treatment device (MQD-50, Jianda Drying Equipment Co., Changzhou, China) as described by Chen et al*.*^22^ at 120 °C until the moisture content was below 3%. Then, the PP, WF, and MAPP were blended in different mass fraction ratios (Table 1) with a high-speed mixer (SHR-10A; Zhangjiagang Tonghe Plastic Machinery Co., Zhangjiagang, China). The mixtures were pelletized with a co-rotating twin-screw extruder (JSH30; Nanjing Rubber and Plastics Machinery, Nanjing, China) and cut in small granules using a pulverizer. The granules were extruded in the WF/PP composite boards with a thickness of 4 mm and width of 100 mm using another single-screw extruder (SJ45; Nanjing Rubber and Plastics Machinery, Nanjing, China). As shown in Table 1, three WF-to-PP ratios were designed.

**Table 1:** Mass ratios of the WF/PP composites.

Composite	Wood Fiber	PP	MAPP
WF/PP = 6/4	60	40	2
WF/PP = 7/3	70	30	1.5
WF/PP = 8/2	80	20	1

### Preparation of the CPP films

**Figure 1:**
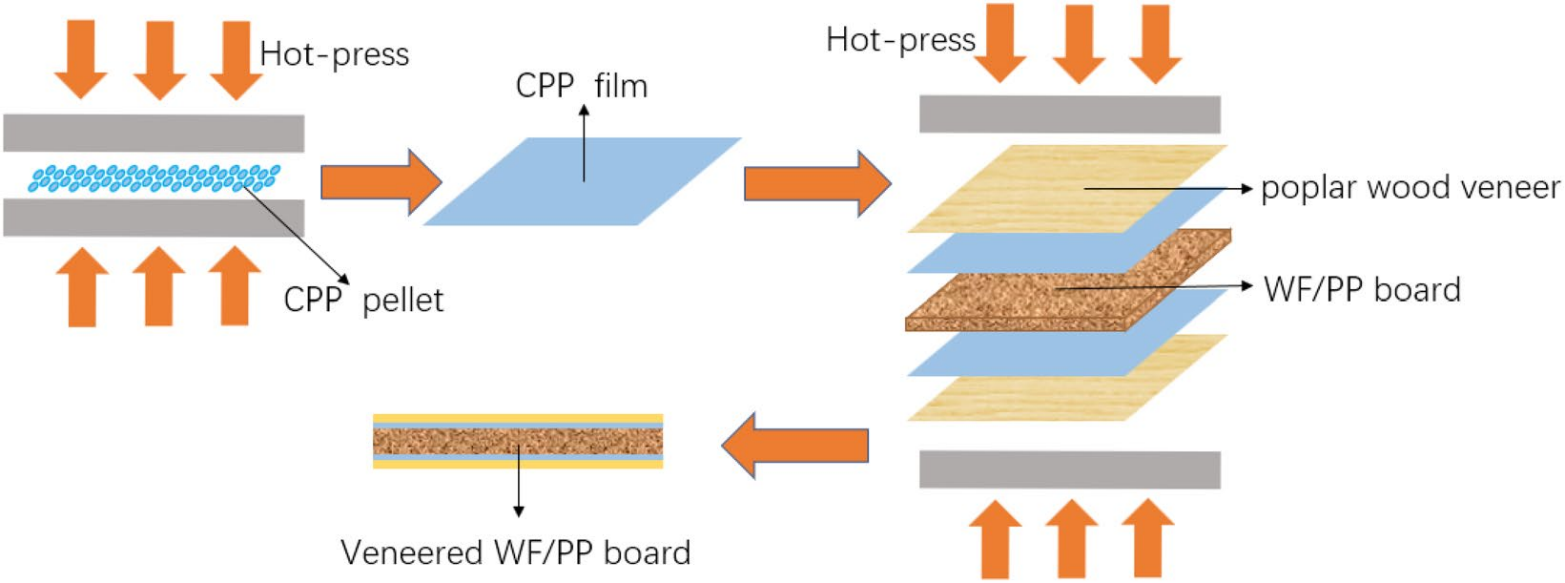
Preparation of veneered WF/PP board with CPP film.

The preparation of the CPP films is shown in Fig. 1. The CPP pellets were evenly spread in a square mold with an inside length of 160 mm and a thickness of 0.1 mm. Then, the mold was hot pressed at 110 °C for 3 min at a pressure of 2 MPa. After hot pressing, the mold was cold pressed for 5 min to cool down to room temperature (20 ± 3 °C). The mold was removed and the CPP was shaped into a square film with a length of 160 ± 1 mm and a thickness of 0.1 ± 0.02 mm.

### Preparation of the veneered WF/PP composite

The CPP film was placed on the surface of the WF/PP composite board and the poplar veneer was then placed upon it, as shown in Fig. 1. The resulting sandwich structure was then pressed at 5 MPa for 5 min at 110 °C and was then allowed to cool in air. The veneered WF/PP boards were then conditioned at 20 °C and 65% relative humidity for 7 days before they were tested.

### Surface bonding strength test

**Figure 2:**
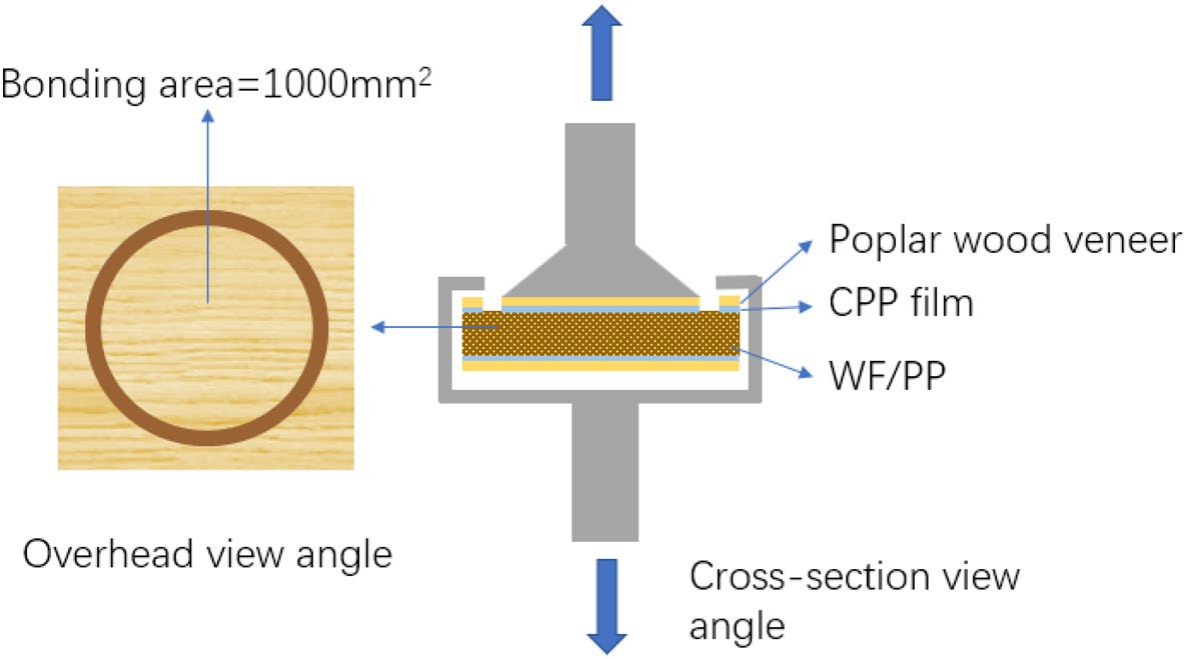
Bonding strength test of the veneered WF/PP composite was measured.

The surface bonding strength of the veneered WF/PP board was tested in accordance with the vertical drawing method, as shown in Fig. 2. The samples had dimensions of 50 mm × 50 mm × 6 mm (length × width × thickness). A circle with an area of 1000 mm^2^ was isolated in the middle of each sample surface by cutting through the veneer and the bonding layer. The isolated circle wood veneer was bonded to the upward fix head with polyurethane hot melt adhesive3731 (Minnesota Mining and Manufacturing Corporation (3 M), Shanghai, China). Then the remainder of the sample was held down by its edges. There were 12 specimens in each group, and the loading rate was 2 mm/s. The experimental instrument was a universal mechanical testing machine (RGT-20A; Shenzhen Reger Instrument Co., Shenzhen, China).

### Surface roughness test

The surface roughness of WF/PP base board was detected with a contact-type surface roughness measuring instrument (SJ-210, Mitutoyo Japan Corporation, Kawasaki, Japan). Maximum height of the profile (Rz), contour arithmetic mean deviation (Ra), surface profile height Root-Mean-Square (Rq) and the Surface contour curve are measured and calculated according to “ISO 4287:1997 Geometrical Product Specifications (GPS)—Surface texture: Profile method—Terms, definitions and surface texture parameters”. Testing length is 5 mm; the probe is made of adamas and movement speed is 0.5 mm/s; the pressure of probe is 4mN; the filter is GAUSS; the number of data collection is 8000.

### Tension property test

**Figure 3:**
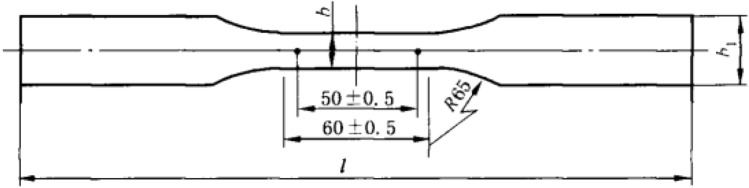
The tension properties test specimen.

The specimens were cut into the shape shown in Fig. 3 and immobilized between two grips. The section width was labeled as “b”, and the grip width was labeled as “b_1_”. The loading rate was 2 mm/min, the distance between the grips was 120 mm, and the specimen thickness was 6 mm. The specimens were tested with the RGT-20A universal mechanical testing machine (Shenzhen, China).

### Flexural property tests

**Figure 4:**
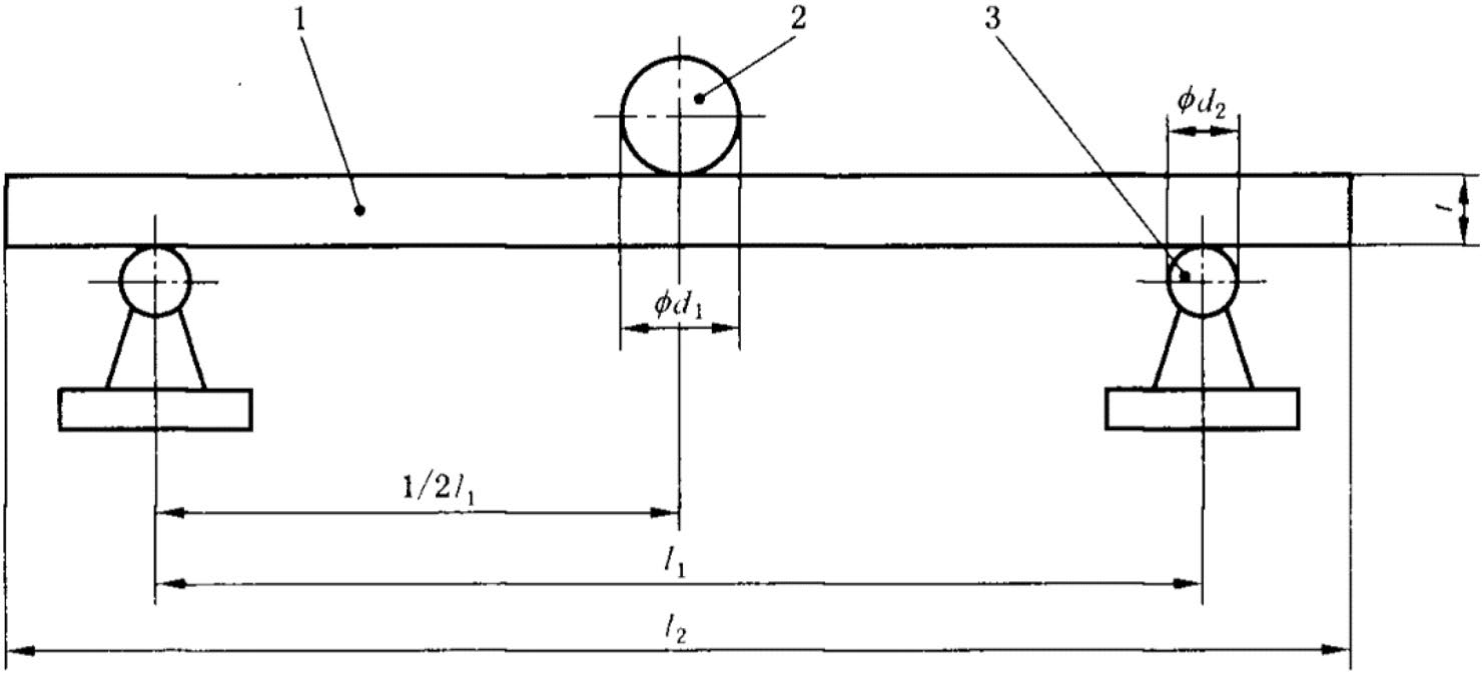
The flexural properties test.

As shown in Fig. 4, the distance (l_1_) between the two supporting heads was 20 times the specimen thickness (l), the diameter of the supporting heads (d_2_) was 15 mm, the specimen width was 50 ± 1 mm, the specimen length (l_2_) was equal to l_1_ plus 50 mm, the loading rate was 10 mm/min, and the diameter of the loading head (d_1_) was 30 mm.

### Low-velocity impact property test

**Figure 5:**
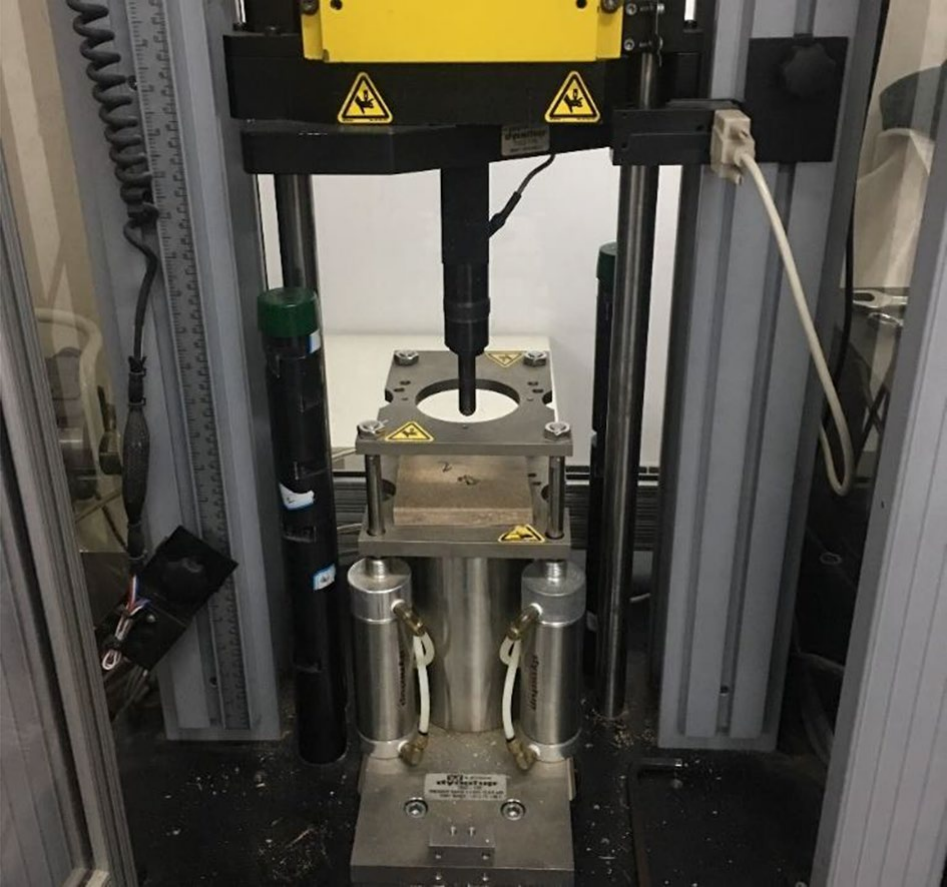
The low-velocity impact test apparatus.

An Instron 9250HV (Norwood, MA, USA) full digital low-velocity impact tester was used to test the low-velocity impact strength, as shown in Fig. 5. The sample was placed in the sample stand to immobilize it. The beam was then adjusted to move the hammer downward until it lightly touched the sample surface. The hammer head was a steel hemisphere with a diameter of 22 mm. The hammer weighed 5.375 kg. Each group of samples was tested with an initial impact energy of 100 J to confirm that the apparatus was able to test them to destruction. In this first trial, all the plates were broken through and exhibited permanent plastic deformation in other ways as well.

The initial impact velocity was 2.156 m/s. The kinetic energy of impact was determined by Eq. (1),

1 $${U}_{0}=\left(\frac{1}{2}\right)m{v}_{0}^{2}$$

where *m* is the hammer mass (kg), *v*_*0*_ is the instantaneous impact velocity when the hammer strikes (m/s), and *U* is the impact energy absorbed by the panel (J). The impact energy absorbed by the panel was calculated according to Eq. (2),

2 $$U=\left(\frac{1}{2}\right)m({v}_{0}^{2}-{v}_{t}^{2})$$

where ${v}_{t}^{2}$ is the maximum instantaneous rebound velocity of the drop hammer (m/s) and $\left(\frac{1}{2}\right)m{v}_{t}^{2}$ is drop hammer kinetic energy that was caused by the elastic deformation energy release of panel (J). The impact load was calculated by Eq. (3),

3 $${F}_{(t)}=m{a}_{\left(t\right)}=md{V}_{(t)}/dt$$

where *a*_*(t)*_ is the instantaneous acceleration (m^2^/s) and *V*_*(t)*_ is the instantaneous velocity during the experiment process (m/s). The displacement during the impact process was calculated by Eq. (4),

4 $${D}_{(t)}={\int }_{0}^{t}{V}_{(t)}dt$$

where *D*_*(t)*_ is the displacement during the impact process (m)^23,24^.

### The scanning electron microscopy (SEM) analysis

The bond layer between the WF/PP composite board and the poplar veneer was observed under a scanning electron microscope (JSM7500F; JEOL, Tokyo, Japan). The section slices were prepared by microtomy (described below). The samples were coated with gold and then examined with an accelerating voltage of 5 kV. The cross-section of the decorated WF/PP composites were investigated by SEM.

## Results and discussion

### Surface bonding strength

**Figure 6:**
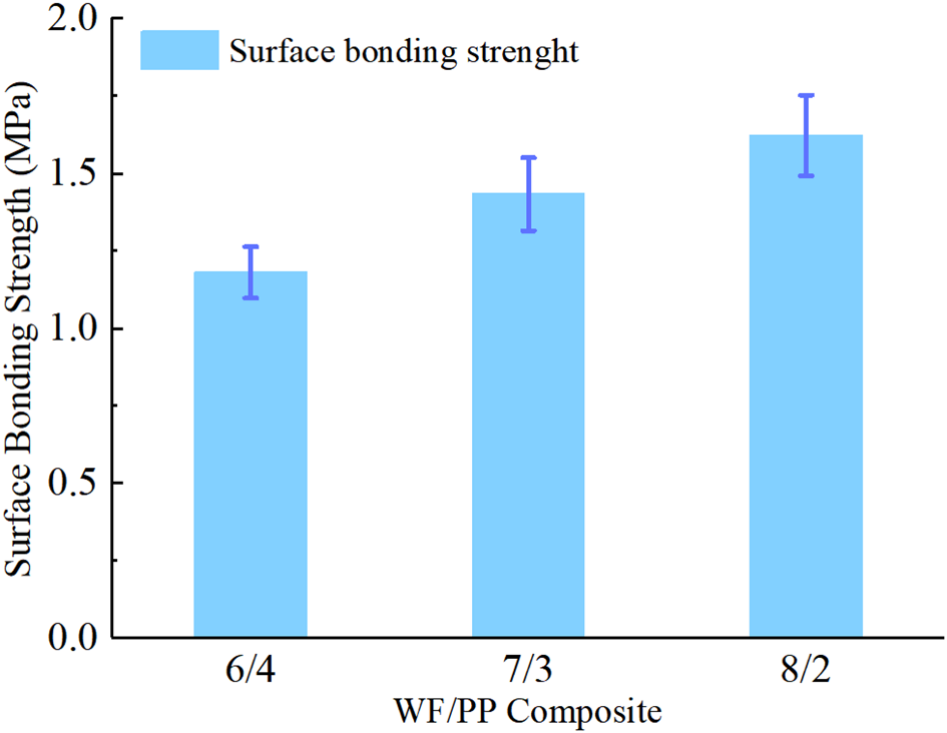
The surface bonding strength of the wood veneered WF/PP composites.

Table 2 shows the arithmetic mean of surface bonding strength of the poplar veneer decorated WF/PP composites with different wood fiber contents. The surface bonding strength of the decorated panel with CPP as the intermediate layer was greater than 1.2 MPa. As the wood fiber content in the WF/PP composite substrate increased, the surface bonding strength of the veneer also increased (Fig. 6). This is because higher wood fiber content increases the surface roughness of the substrate, which in turn increases the microscopic surface area and thereby improves bonding.

**Table 2:** Surface bonding strength (MPa).

WF/PP	6/4	7/3	8/2
Bonding strength	1.18	1.43	1.62

**Figure 7:**
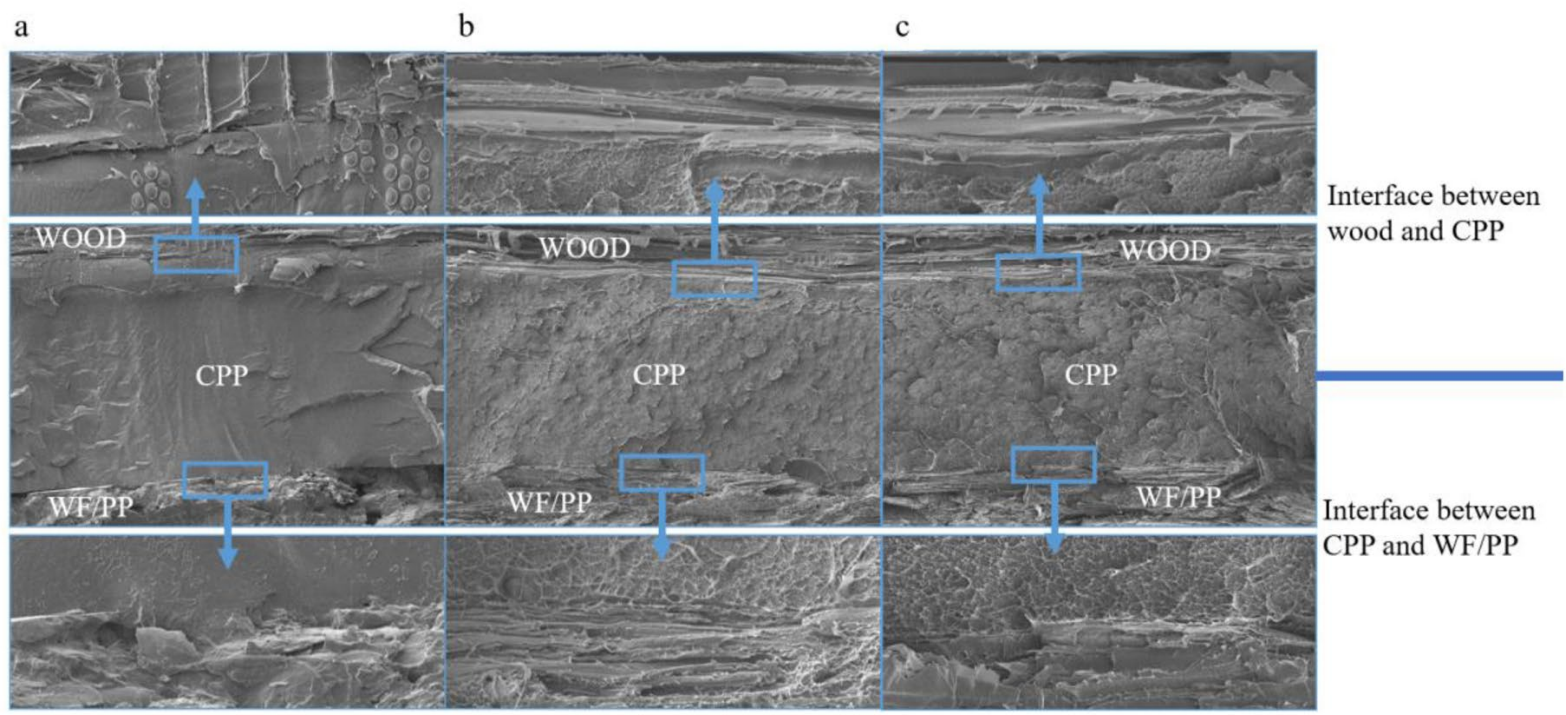
The cross-section SEM images of the interfaces between the wood veneer, CPP, and WF/PP (**a**) WF/PP = 6/4, (**b**) WF/PP = 7/3, and (**c**) WF/PP = 8/2 composite boards.

The cross-section SEM images of the WF/PP composites is shown in Fig. 7. The three layers of WF/PP, CPP, and poplar veneer were distinctly apparent. There was a small gap between the CPP and WF/PP base material when the ratio of WF to PP was 6/4 (Fig. 7a). The gap became thinner with as the wood fiber content increased, as seen in Fig. 7b,c.

### Surface roughness

**Figure 8:**
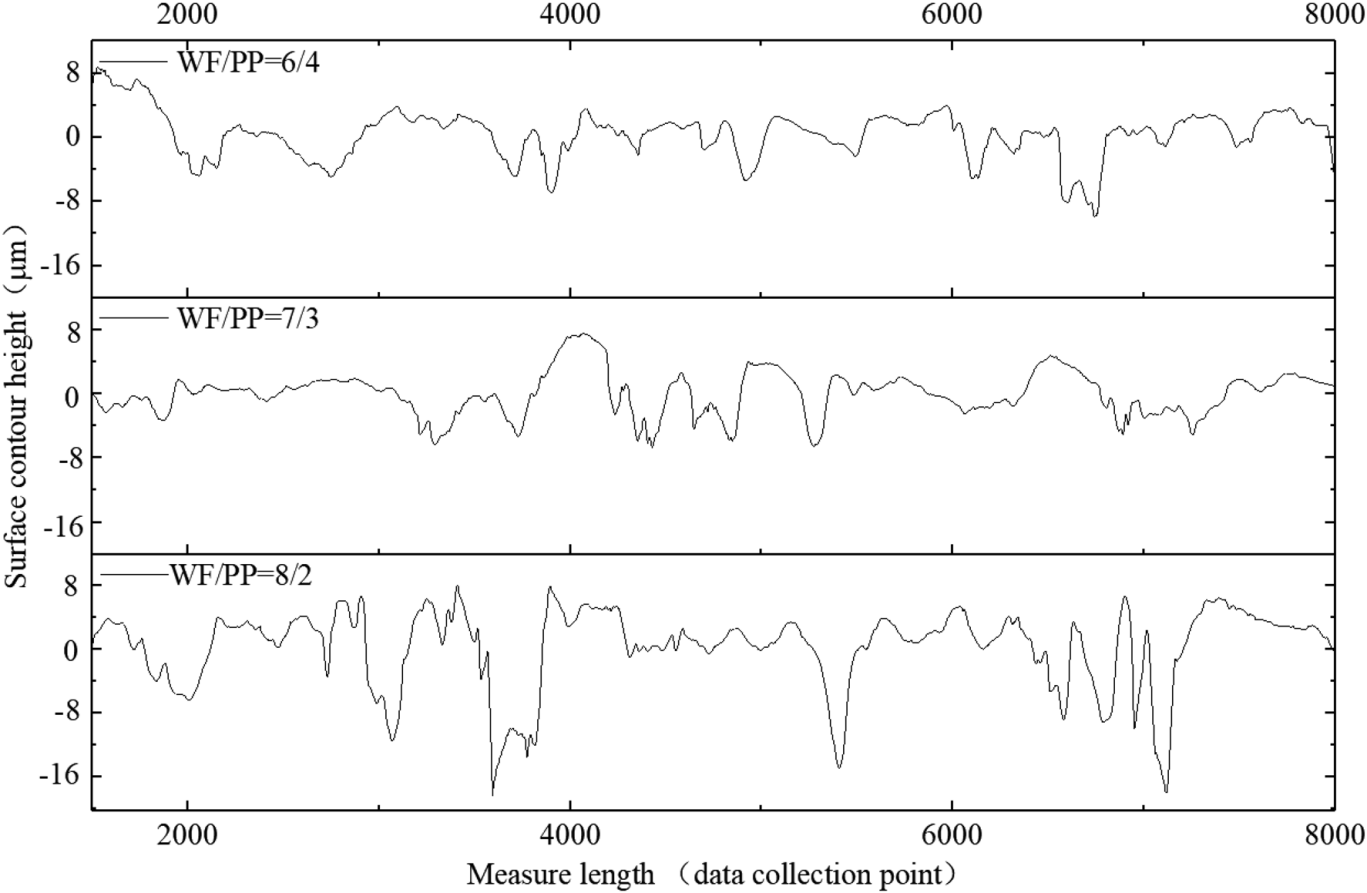
Surface contour of WF/PP composite.

The surface roughness is one of factors effecting bonding strength^25,26^. It is found in Fig. 8 that the surface contour height rangeability is raising with the increasing of wood fiber content in WF/PP composite. Those dents could provide more specific surface area and embedded point for the molten CPP. Parameters of surface roughness are calculated on the basis of ISO 4287:1997 and listed in Table 3. Ra and Rq represent the surface roughness and higher value, respectively, which means the larger roughness. The Rz means the maximum height from the lowest point to the highest. The parameter Ra, Rq and Rz all increase with the increasing of wood fiber content. Combining the statement in surface bonding strength, this surface roughness is owing to the exposed wood fibers. Those exposed wood fibers could not form a smooth surface like PP. The surface roughness is related to the bonding strength of veneered WF/PP composite board.

**Table 3:** Surface roughness of WF/PP base board.

WF/PP composite	Ra (μm)	Rz (μm)	Rq (μm)
60WF/40PP	1.595	9.389	2.108
70WF/30PP	2.117	13.601	2.713
80WF/20PP	3.409	18.914	4.231

### Tension properties

**Figure 9:**
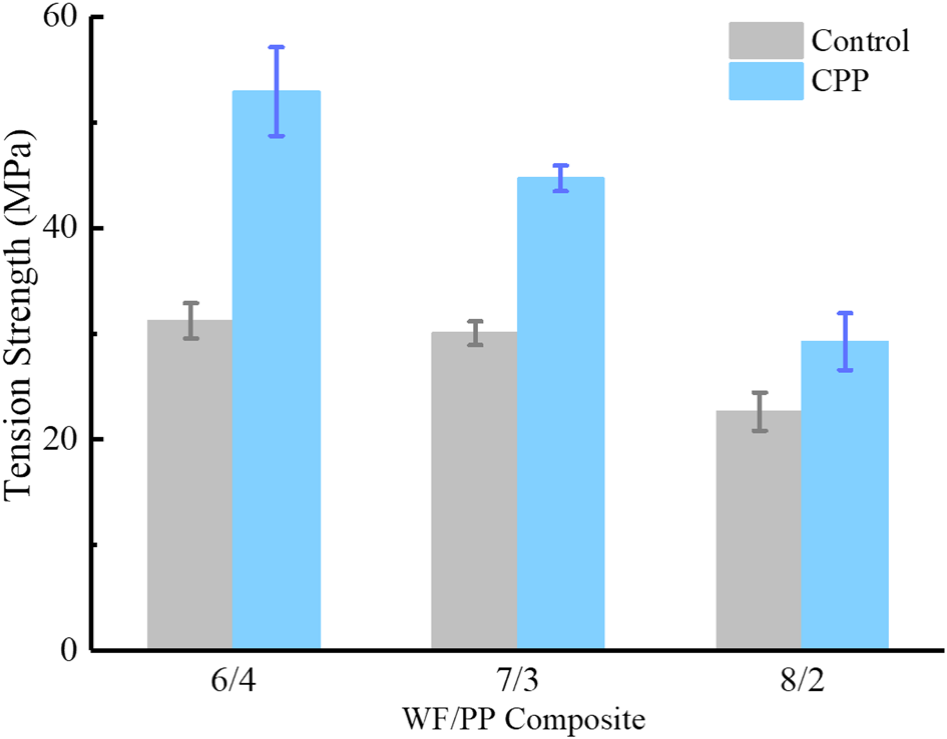
The tension strength properties of the WF/PP composites.

As shown in Fig. 9 and Table 4, the tensional strength of the control group and the veneered WF/PP composite group declined as the wood fiber content increased. This is because wood fiber disrupts the continuity of the WF/PP composite system at high wood fiber loadings. The PP matrix was unable to completely cover and bond to the wood fibers in the high wood fiber content composites, which reduced the composite tensional strength. The addition of veneers enhanced the tensional strength of the composite.

**Table 4:** Tension properties of WF/PP composite board.

Wood fiber content	WF/PP = 6/4		WF/PP = 7/3		WF/PP = 8/2	
Control/Veneer	Control	CPP	Control	CPP	Control	CPP
Tension strength (MPa)	31.19	52.90	30.02	44.66	22.65	29.22
Tension modulus (GPa)	1.91	2.55	2.05	2.57	2.31	2.50

**Figure 10:**
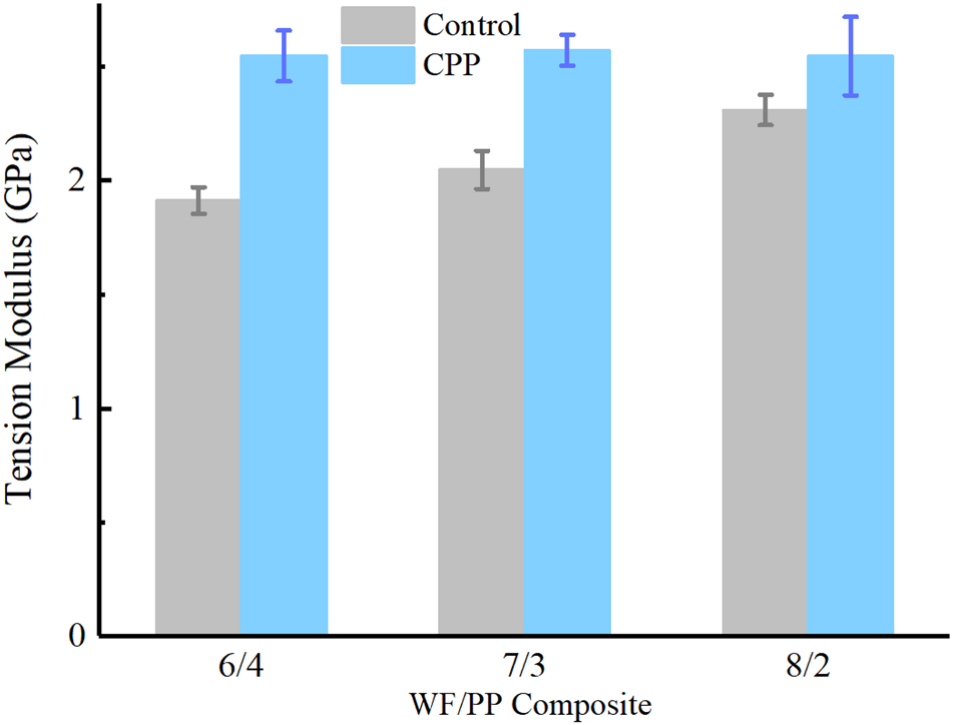
The tension modulus properties of the WF/PP composites.

However, the tensional modulus followed the opposite trend (Fig. 10). The tensile deformation resistance of the WF/PP composites increased as the wood fiber content increased, which indicated that the wood fiber in the composite enhanced the tension modulus of the composite. The average value of tension modulus of the veneered composites is over 2.5 GPa. The wood veneer increased the tension modulus and appeared to have been the primary factor that determined the tension modulus in all the veneered samples because all the WF/PP ratios afforded a similar tension modulus once veneered within the margin of error.

### Flexural properties

**Figure 11:**
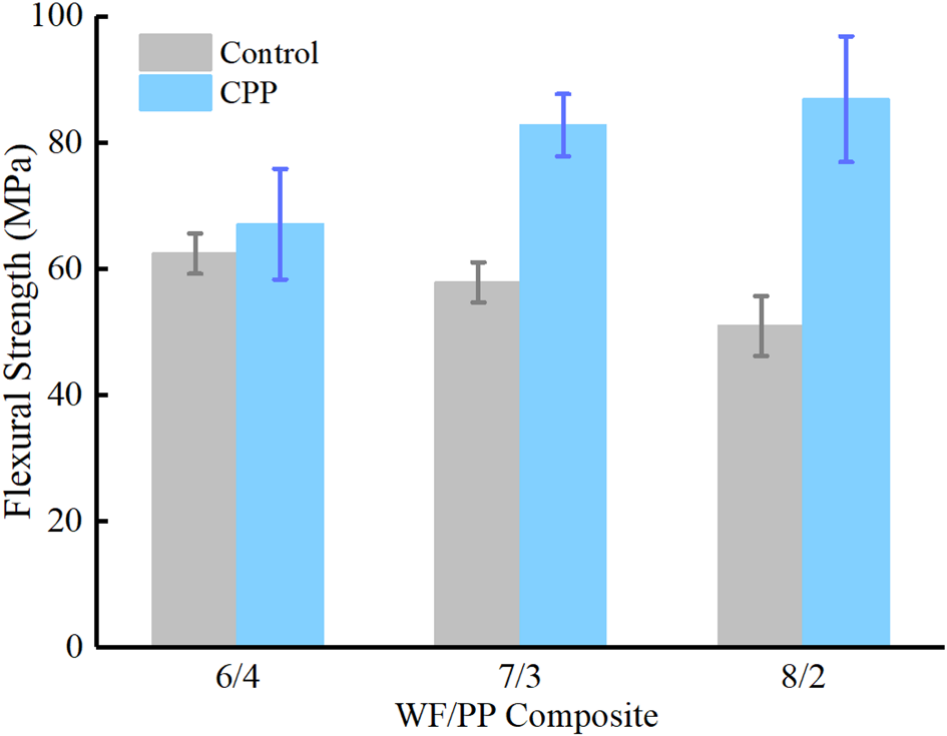
The flexural strength properties of the WF/PP composites.

The flexural strength results are shown in Fig. 11 and Table 5. In the control group, the flexural strength is similar to the data in Chattopadhyay’s report^27^. The flexural strength of control group declined as the wood fiber content increased. As with the reported tension test results, the addition of wood fiber would be expected to diminish the flexural strength. However, the veneers produced a remarkable increase in flexural strength in ways that once more dominated the results. This was to be expected as the flexural strength of a sample in bending is dominated by the mechanical properties of the layer furthest from its bending centerline. The flexural strength decreased as the wood fiber content in the WF/PP composites increased. The starkly opposite trend of flexural strength between the control group and the decorated group was attributed to the strength of the wood veneers and the effectiveness of their bonding to the WF/PP composite by way of the CPP interface. This became more effective as the wood fiber content in the WF/PP composites increased, as shown in Fig. 6.

**Table 5:** Flexural properties of WF/PP composite board.

Wood fiber content	WF/PP = 6/4		WF/PP = 7/3		WF/PP = 8/2	
Control/veneer	Control	CPP	Control	CPP	Control	CPP
Flexural strength (MPa)	62.4	67.10	57.84	82.7	50.94	86.85
Flexural modulus (GPa)	4.58	6.59	5.51	7.30	6.77	8.10

**Figure 12:**
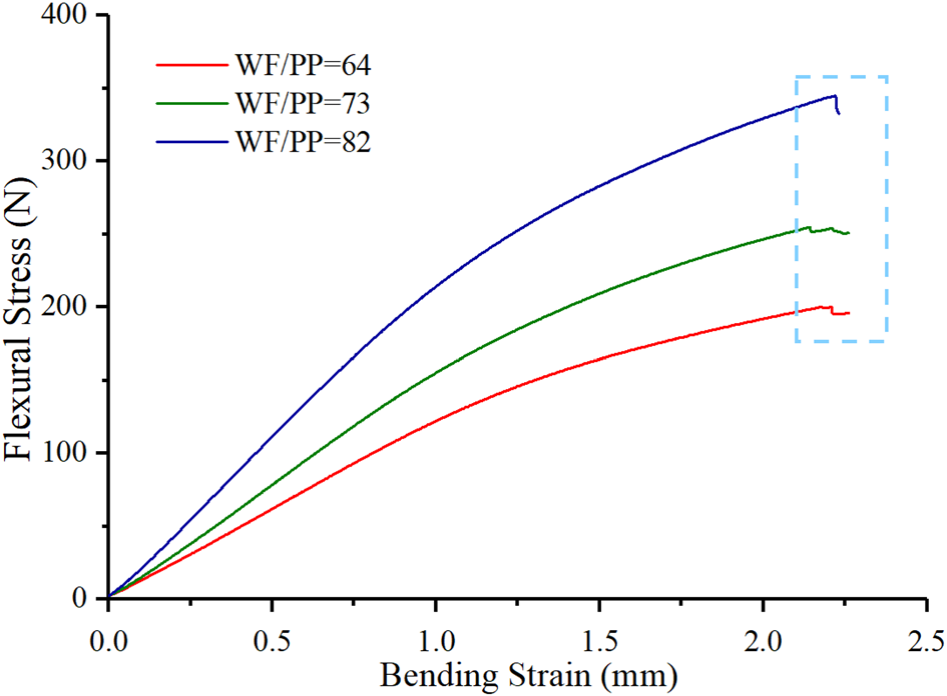
The bending strain versus the flexural stress of the veneered WF/PP composites.

The flexural stress-bending strain curve, shown in Fig. 12, clarified the bonding performance between the WF/PP composite and the CPP and its effect on the flexural strength. The bending strain increased as the flexural stress increased, but the curves presented a different shape near the failure point. The WF/PP = 6/4 and WF/PP = 7/3 specimens had a stress relaxation that indicated that the failure of these two veneered composites was layer by layer. As stress increases, the strain between each layer would increase the interlaminar shear, and the bond between the CPP and the WF/PP may break. In such an event, the failure is indicated in the flexural stress-bending strain curve as a stepping-down of the load. The curve of the WF/PP = 8/2 composite showed no step phase, only an abrupt loss of load. This indicated that the interface between the WF/PP and the CPP was firm, and therefore failure occurred as tensile rupture rather than interlaminar shear failure.

**Figure 13:**
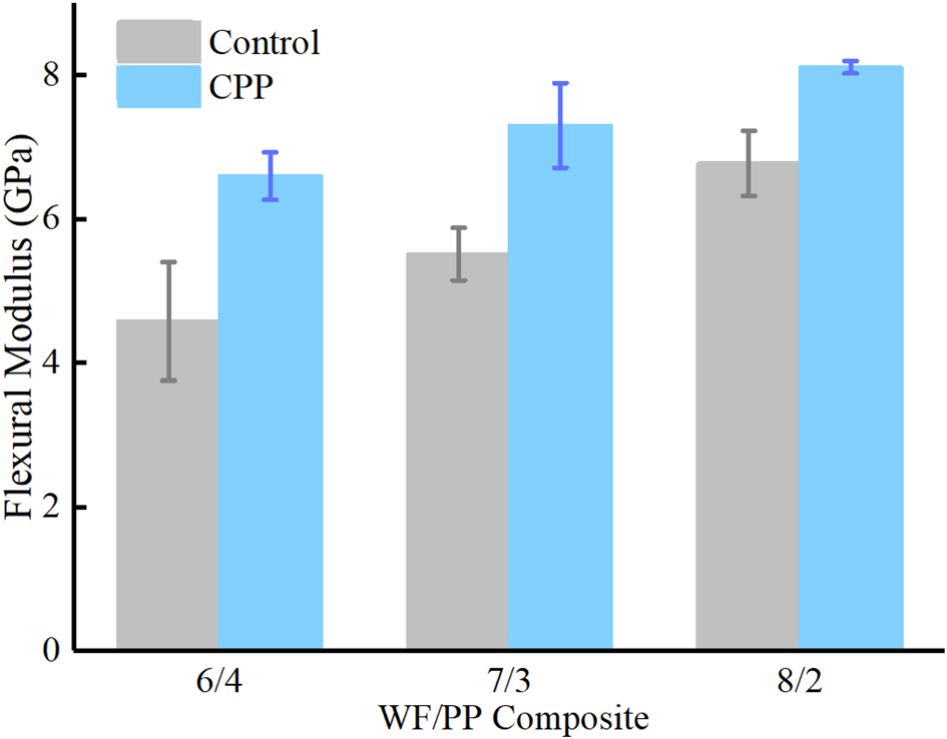
The bending modulus of the WF/PP composites.

The flexural modulus of the control group and the veneered composite both increased as the wood fiber content increased (Fig. 13). The tensional modulus also increased as the wood fiber content increased because the wood fibers have a higher tensional modulus than the PP. In addition, in compression the wood fibers are directionally aligned in the WF/PP composite in the direction of extrusion. The bending stiffness of the veneered WF/PP composite was enhanced by the wood fibers inside the base material and by the veneer outside the WF/PP composite.

### Low-velocity impact properties

**Figure 14:**
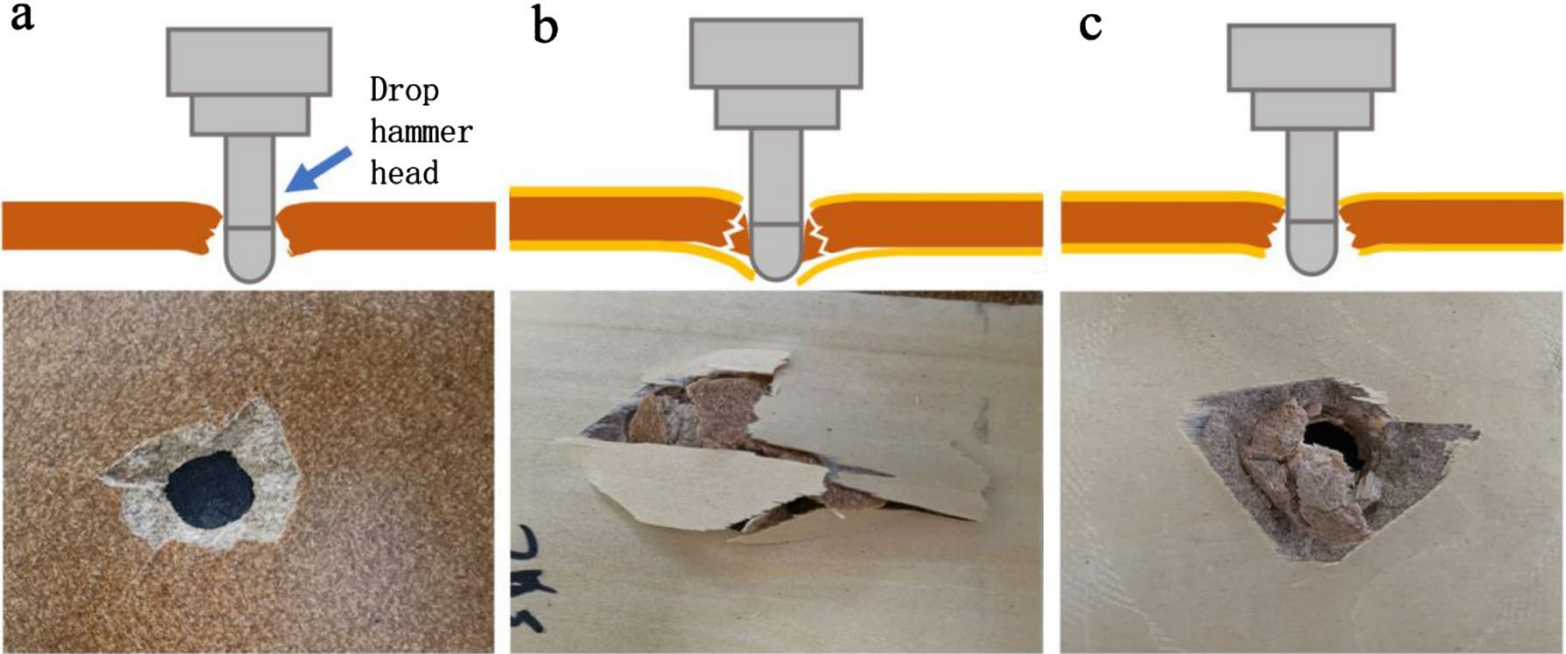
Images of the typical perforation forms of the (**a**) WF/PP = 6/4 (control), (**b**) WF/PP = 6/4, and (**c**) WF/PP = 8/2 composite boards after the low-velocity impact testing.

Figure 14a shows the perforated underside of the WF/PP = 6/4 composite from the unveneered control group after the impact of the drop hammer at 100 J. As the diagram shows, the hole was roughly circular on the impact side and there was evidence of a fully departed shatter cone on the underside. Figure 14b,c show the perforated undersides of the veneered WF/PP composite after the low-velocity impact at 100 J. The impact face of these specimens had a small, neat hole, like that of the control WF/PP in Fig. 14a. However, the underside surface of the veneered composite showed different destruction patterns with different WF/PP ratios. The underside wood veneer of the WF/PP = 6/4 composite split from the WF/PP composite base material. This confirmed that after the drop hammer head perforated the WF/PP composite, the hammer head was separated the wood veneer from the WF/PP composite before it penetrated the wood veneer in a separate and secondary action. The underside destruction of the WF/PP = 8/2 composite displayed a tidy edge, a pattern that was very different from the WF/PP = 6/4 specimen. The result in Fig. 14c shows that the wood veneer and WF/PP composite cohered tightly as a whole plate right through the impact, with the veneer and WF/PP remaining bonded.

**Figure 15:**
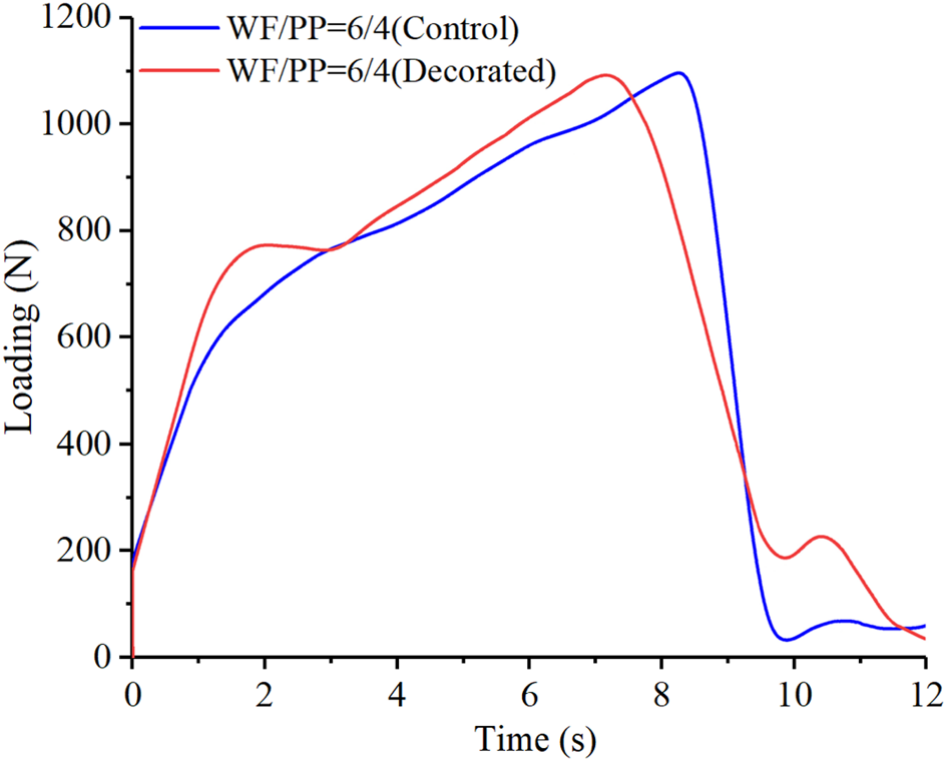
The low-velocity impact loading-time curve of the WF/PP = 6/4 specimens.

**Figure 16:**
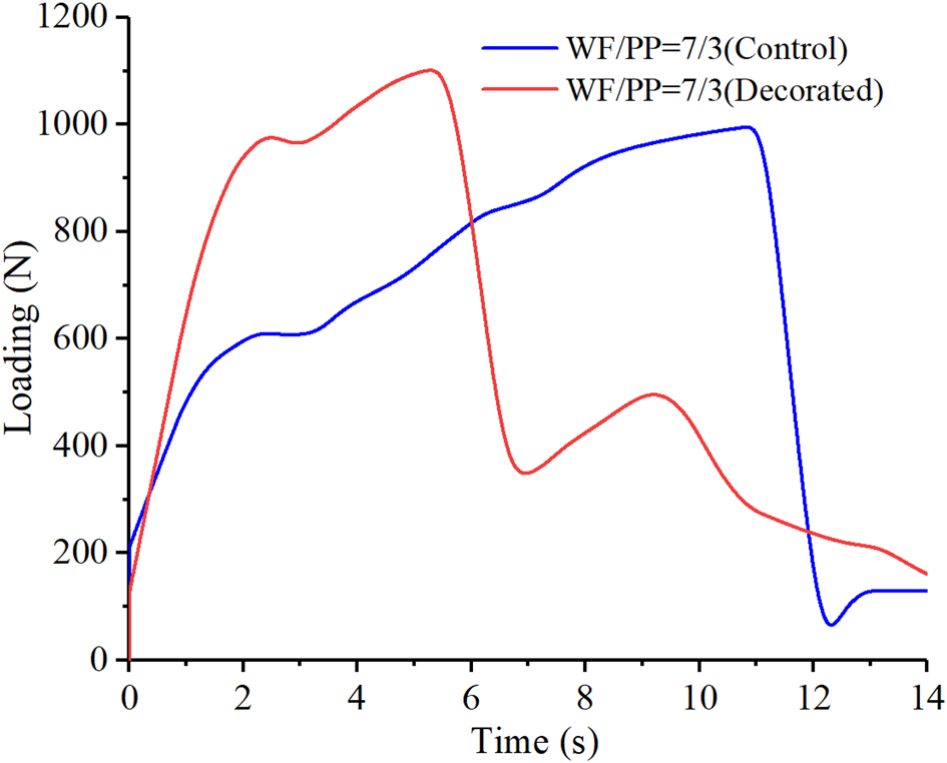
The low-velocity impact loading-time curve of the WF/PP = 7/3 specimens.

**Figure 17:**
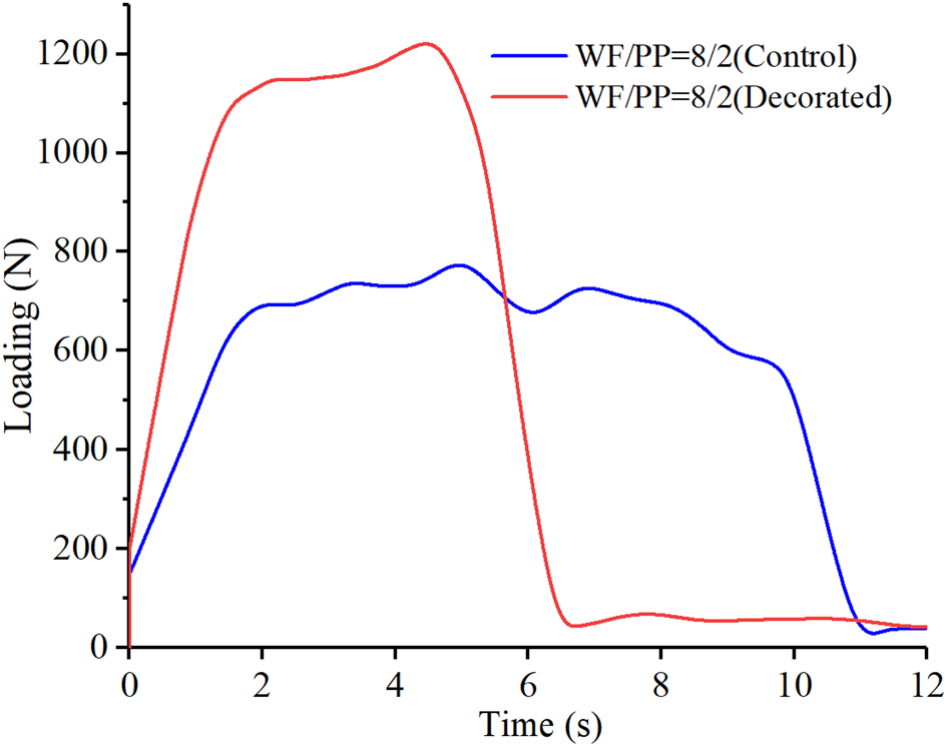
The low-velocity impact loading-time curve of the WF/PP = 8/2 specimens.

The impact loading-time results of three compositions are shown in Figs. 15, 16, and 17. In the initial phase of the test (approximately 2 s in duration), the loading rapidly increased in a linear relation with time. Both the control and the veneered samples showed this relation across all three compositions. Once the wood veneer was broken through, which happened after approximately 2 s, the load exhibited a small decline. Soon thereafter, the resistance increased due to contact with the WF/PP composite core. The veneered specimens behaved similarly to the controls during this phase of the test. The control group and veneered group reached the impact loading peak at nearly the same time and at almost exactly the same applied force with the WF/PP = 6/4 specimen. When the interface cross-section morphology of Fig. 7 and the interface bonding strength of the decorated composite in Fig. 6 are jointly considered, it appears that the wood veneer failed to enhance the composite board penetration resistance with the WF/PP = 6/4 because of the weak bonding strength between the base board surface and the wood veneer. However, the decorated composite impact loading peak force was higher than that of the control group with the WF/PP = 7/3 and WF/PP = 8/2 specimens. This was attributed to the interface bonding strength between the WF/PP core and the wood veneer, which was sufficient to make the veneered panel behave as a whole panel for this test. This suggests the enhancement effect of wood veneer with regards to penetration is strongly influenced by the interface bonding strength.

**Figure 18:**
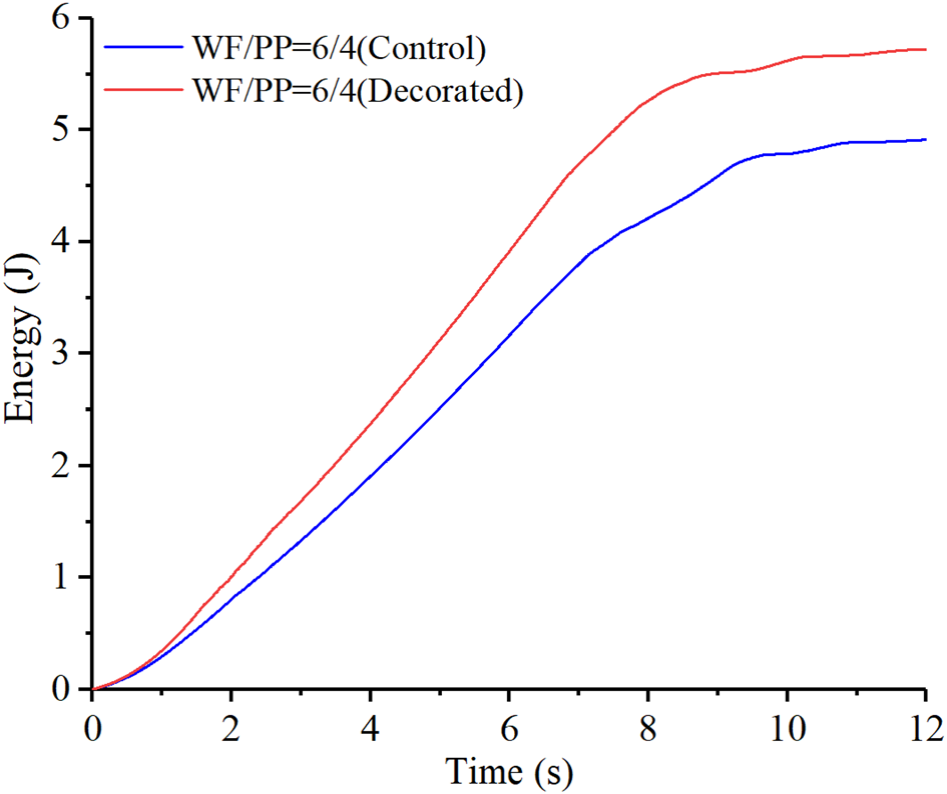
The drop hammer strain energy-time curve of the WF/PP = 6/4 specimen.

**Figure 19:**
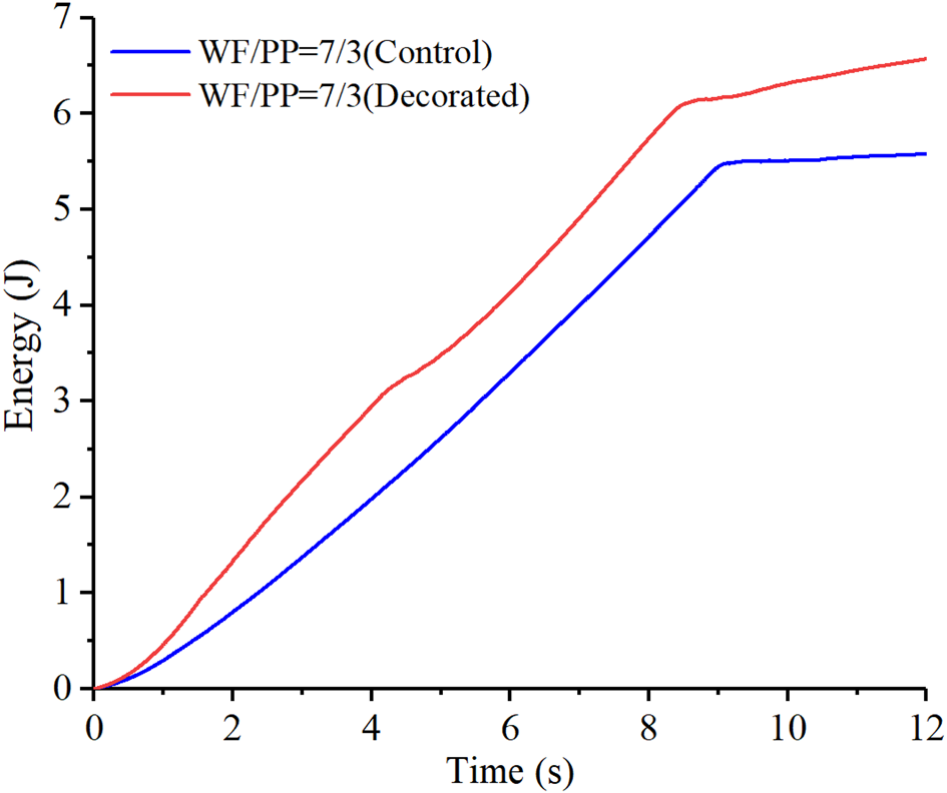
The drop hammer strain energy-time curve of the WF/PP = 7/3 specimen.

**Figure 20:**
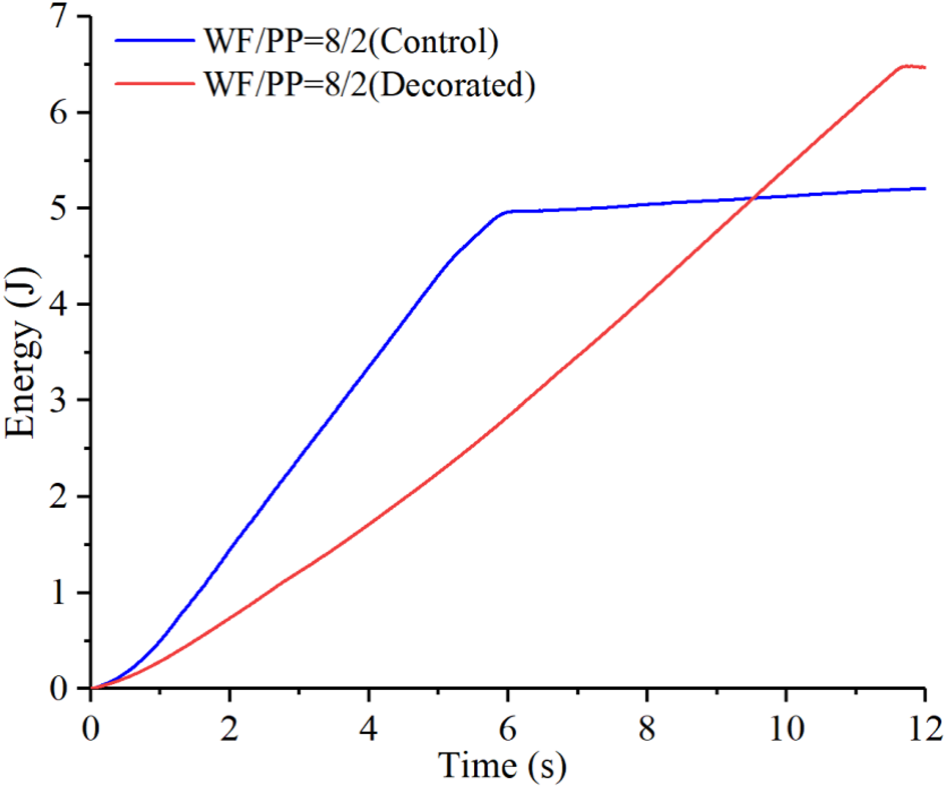
The drop hammer strain energy-time curve of the WF/PP = 8/2 specimen.

As shown in Figs. 18, 19, and 20, the elastic work plays a decisive role in the initial stage of energy absorption. Plastic deformation work and crack propagation absorb further energy slightly before the material begins to break in massive terms. As the impact cracks grow, the material in the impact region ultimately breaks instantly. At this moment, the energy rises rapidly. When the impact head pierces the entire composite board, the energy curve tends to level off.

The strain energy to break through the decorated WF/PP composite board was higher than the control group. This is because the wood veneer was softer than the WF/PP composite board, so a part of the strain energy was absorbed by the wood veneer. The elastic adhesive layer would also absorb some of the strain energy. On the other hand, the wood veneer reinforced the whole composite board, which resulted in a higher energy absorption. The WF/PP = 8/2 veneered specimen had the highest interface bonding strength and the WF/PP = 6/4 veneered specimen had the lowest. However, both the WF/PP = 7/3 and WF/PP = 8/2 veneered composites appeared to absorb one more joule of energy in this test than the WF/PP = 6/4 veneered composite.

## Conclusions

1. The surface bonding strength of veneered WF/PP composites with CPP as a bonding layer exceeded 1.2 MPa. As the wood fiber content in the WF/PP increased, the bonding strength between the wood veneer and the WF/PP composite also increased. The interface between the CPP and WF/PP also tended to close.
2. The wood veneer enhanced the tensile strength of the composite. The wood veneer outside the composite also increased the tension modulus.
3. The flexural strength and modulus were both enhanced by the wood veneer. The bending properties were also influenced by the strength of the interface between the wood veneer, the CPP layer, and the WF/PP composite core.
4. The low-velocity impact and progressive penetration test results indicated that a higher wood fiber content of the WF/PP base board and a higher surface bonding strength will each lead to higher impact resistance and energy absorption.
5. The wood veneered WF/PP composites board with CPP as adhesive have a high mechanical property and graceful appearance which is appropriate for being the floor indoor and other indoor furniture materials.

## References

[1] Ashori A, Nourbakhsh A (2009). Characteristics of wood-fiber plastic composites made of recycled materials. Waste Manag..

[2] Niska KO, Sain M (2008). Wood-Polymer Composites.

[3] Selke SE, Wichman I (2004). Wood fiber/polyolefin composites. Compos. A Appl. Sci. Manuf..

[4] Nourbakhsh A, Ashori A (2009). Preparation and properties of wood plastic composites made of recycled high-density polyethylene. J. Compos. Mater..

[5] Turku I, Keskisaari A, Kärki T, Puurtinen A, Marttila P (2017). Characterization of wood plastic composites manufactured from recycled plastic blends. Compos. Struct..

[6] Sommerhuber PF, Wang T, Krause A (2016). Wood-plastic composites as potential applications of recycled plastics of electronic waste and recycled particleboard. J. Clean. Prod..

[7] Schwarzkopf, M. J. & Burnard, M. D. Wood-plastic composites—performance and environmental impacts. In *Environmental Impacts of Traditional and Innovative Forest-Based Bioproducts* 19–43 (Springer, 2016).

[8] Kinoshita H, Kaizu K, Fukuda M, Tokunaga H, Koga K, Ikeda K (2009). Development of green composite consists of woodchips, bamboo fibers and biodegradable adhesive. Compos. B Eng..

[9] Pickering KL, Efendy MGA, Le TM (2016). A review of recent developments in natural fibre composites and their mechanical performance. Compos. A Appl. Sci. Manuf..

[10] Mazzanti V, Mollica F, El Kissi N (2016). Rheological and mechanical characterization of polypropylene-based wood plastic composites. Polym. Compos..

[11] Smith PM, Wolcott MP (2006). Opportunities for wood/natural fiber-plastic composites residential and industrial applications. For. Prod. Soc..

[12] Bledzki A, Gassan J, Theis S (1998). Wood-filled thermoplastic composites. Mech. Compos. Mater..

[13] Cholake ST, Rajarao R, Henderson P, Rajagopal RR, Sahajwalla V (2017). Composite panels obtained from automotive waste plastics and agricultural macadamia shell waste. J. Clean. Prod..

[14] Teuber L, Osburg VS, Toporowski W, Militz H, Krause A (2016). Wood polymer composites and their contribution to cascading utilisation. J. Clean. Prod..

[15] Ashori A (2008). Wood-plastic composites as promising green-composites for automotive industries!. Bioresour. Technol..

[16] Golmakani ME, Wiczenbach T, Malikan M, Mahoori SM, Eremeyev VA (2021). Experimental and numerical investigation of tensile and flexural behavior of nanoclay wood-plastic composite. Materials.

[17] Friedrich D (2021). Thermoplastic moulding of Wood-Polymer Composites (WPC): A review on physical and mechanical behaviour under hot-pressing technique. Compos. Struct..

[18] Gupta BS, Reiniati I, Laborie M-PG (2007). Surface properties and adhesion of wood fiber reinforced thermoplastic composites. Colloids Surf. A.

[19] Dubreuil M, Bongaers E (2008). Use of atmospheric pressure plasma technology for durable hydrophilicity enhancement of polymeric substrates. Surf. Coat. Technol..

[20] Put S, Bertels C, Vanhulsel A (2013). Atmospheric pressure plasma treatment of polymeric powders. Surf. Coat. Technol..

[21] Liu Y, Sun Y (2019). Interface bonding properties and mechanism of poplar board-veneered wood fiber/polypropylene composites with chlorinated polypropylene films as an intermediate layer. Langmuir ACS J. Surf. Colloids.

[22] Chen F, Li Q, Gao X, Han G, Cheng W (2017). Impulse-cyclone drying treatment of poplar wood fibers and its effect on composite material's properties. BioResources.

[23] Richardson MOW, Wisheart MJ (1996). Review of low-velocity impact properties of composite materials. Compos. A Appl. Sci. Manuf..

[24] Lee SM, Zahuta P (1991). Instrumented impact and static indentation of composites. J. Compos. Mater..

[25] Ozcan S, Ozcifci A, Hiziroglu S (2012). Effects of heat treatment and surface roughness on bonding strength. Constr. Build. Mater..

[26] Uehara K, Sakurai M (2002). Bonding strength of adhesives and surface roughness of joined parts. J. Mater. Process. Technol..

[27] Chattopadhyay SK, Khandal RK, Uppaluri R (2010). Bamboo fiber reinforced polypropylene composites and their mechanical, thermal, and morphological properties. J. Appl. Polym. Sci..

